# Nationwide variation in the effects of temperature on infectious gastroenteritis incidence in Japan

**DOI:** 10.1038/srep12932

**Published:** 2015-08-10

**Authors:** Daisuke Onozuka, Akihito Hagihara

**Affiliations:** 1 Department of Health Communication, Kyushu University Graduate School of Medical Sciences, Fukuoka, Japan.

## Abstract

Although several studies have investigated the effects of temperature on the incidence of infectious gastrointestinal disease in a single city or region, few have investigated variations in this association using nationwide data. We obtained weekly data, gathered between 2000 and 2012, pertaining to infectious gastroenteritis cases and weather variability in all 47 Japanese prefectures. A two-stage analysis was used to assess the nonlinear and delayed relationship between temperature and morbidity. In the first stage, a Poisson regression allowing for overdispersion in a distributed lag nonlinear model was used to estimate the prefecture-specific effects of temperature on morbidity. In the second stage, a multivariate meta-analysis was applied to pool estimates at the national level. The pooled overall relative risk (RR) was highest in the 59.9^th^ percentile of temperature (RR, 1.08; 95% CI: 1.01, 1.15). Meta-analysis results also indicated that the estimated pooled RR at lower temperatures (25^th^ percentile) began immediately but did not persist, whereas an identical estimate at a higher temperature (75^th^ percentile) was delayed but persisted for several weeks. Our results suggest that public health strategies aimed at controlling temperature-related infectious gastroenteritis may be more effective when tailored according to region-specific weather conditions.

Infectious gastroenteritis accounts for 1 billion episodes of diarrhea and 3 million deaths in children <5 years of age per year, and it represents the fifth-leading cause of death worldwide^1^,^2^. The transmission of infectious gastroenteritis is complex and multifactorial, involving both host and environmental factors.

Ambient temperature may be important in the spread and seasonality of infectious gastroenteritis^3^,^4^,^5^,^6^,^7^,^8^,^9^. However, the majority of previous studies have examined the effects of temperature only in a single city or region, thus not representing the wide range of climatic, social, demographic, and cultural conditions that occur at the national level. A recent study estimated the potential effects of projected climate change on diarrhea in six regions using five empirical datasets and reported an association between increased diarrhea incidence and higher temperatures^10^. However, that study involved linear assumptions (pertaining to the temperature–diarrhea incidence relationship), and each empirical dataset was associated with only a single geographical region. Furthermore, previous studies have demonstrated distinctly nonlinear relationships between temperature and infectious gastroenteritis incidence, in which the morbidity-modifying effects of temperature were spatially heterogeneous^5^,^6^,^7^,^8^,^9^. However, no studies have assessed a diverse range of communities exposed to a variety of climatic conditions. It is essential to elucidate the complex, nonlinear, and multi-parameter relationship that exists between climate variability and infectious gastroenteritis transmission on a national scale.

Multivariate meta-analysis within distributed lag nonlinear models is useful to estimate and pool nonlinear and delayed associations, using time-series data drawn from multiple locations^11^. This two-stage approach reduces over-simplification of the shape of the exposure–response relationship or lag structure, and may be applied to any context requiring that nonlinear and delayed relationships be assessed within different groups^11^,^12^. The majority of previous studies have used only conventional linear exposure–responses and univariate meta-analysis technqiues, which describe the relationship less completely and may not take account of important variables^13^. Therefore, improved understanding of the sensitivity of the two-stage analysis of climate variability may facilitate the development of a reliable, climate-based system for the predication of infectious gastroenteritis epidemics.

In the present study, we explored variations in the relationship between temperature and weekly infectious gastroenteritis incidence between 2000 and 2012 in all 47 Japanese prefectures. To the best of our knowledge, this is the first report to quantify national variations in the effects of temperature on infectious gastroenteritis incidence using a two-stage, time-series analysis.

## Results

We analyzed a total of 12,915,273 (100%) infectious gastroenteritis cases occurring between 2000 and 2012 among 47 Japanese prefectures. The weekly average number of cases was 406.5 (individual prefecture range: 70.8–1265.6). The weekly mean temperature across the 47 prefectures was 15.5 °C (range: 9.3–23.3 °C), the weekly mean humidity was 68.4% (range: 59.5–77.2%), and the weekly mean rainfall was 4.5 mm (range: 2.6–7.1 mm). Latitude ranged between 26.2 and 43.1 degrees north. The mean number of cases and mean temperature varied markedly by prefecture, consistent with the diverse range of climatic conditions (Table S1).

**Figure 1:**
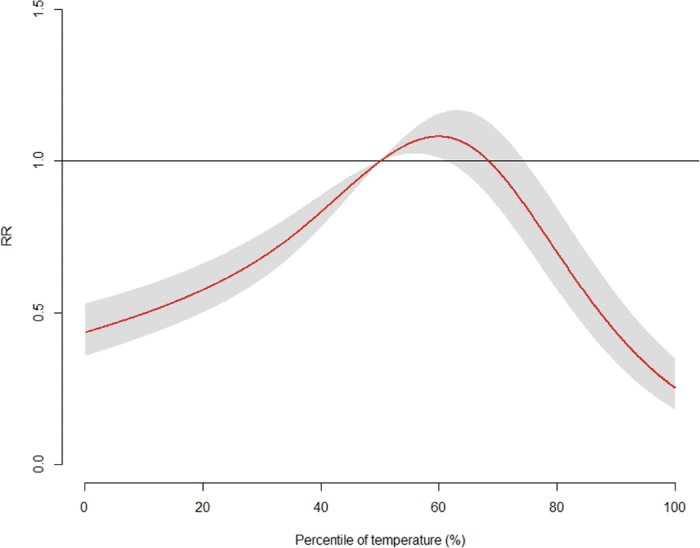
The pooled overall cumulative temperature-morbidity association in all 47 Japanese prefectures. Reference at 50^th^ percentile of temperature.

The pooled overall cumulative relationship between relative risk (RR) of infectious gastroenteritis and temperature, illustrated in Fig. 1, exhibited a broadly inverted U-shape. The highest pooled overall RR was 1.08 (95% CI: 1.01, 1.15) in the 59.9th percentile of temperature. The multivariate Cochran Q test for heterogeneity was significant (*p* < 0.001), and the related *I*^*2*^ statistic indicated that 83.1% of the variability was due to true heterogeneity between prefectures.

**Figure 2:**
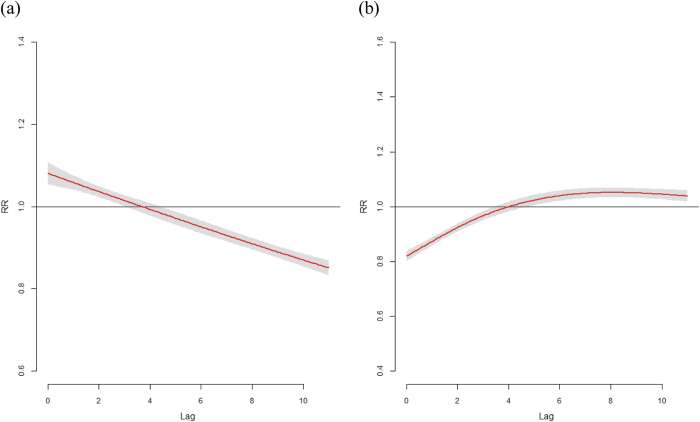
The pooled predictor-specific temperature-morbidity association in all 47 Japanese prefectures. The pooled (95% CI as grey area) summaries at (**a**) 25^th^ and (**b**) 75^th^ percentiles of temperature. Reference at 50^th^ percentile of temperature.

Pooled estimates of predictor-specific summary associations at lower (25^th^ percentile) and higher (75^th^ percentile) temperatures based on the main model are illustrated in Fig. 2. The effects of lower temperature began immediately and disappeared after 2–3 weeks; in contrast, the effects of higher temperatures were delayed and then persisted for several weeks. Cochran Q tests were significant for the lag curve in the 25^th^ and 75^th^ temperature percentiles (*p* < 0.001), with *I*^*2*^ values of 69.5% and 66%, respectively.

**Figure 3:**
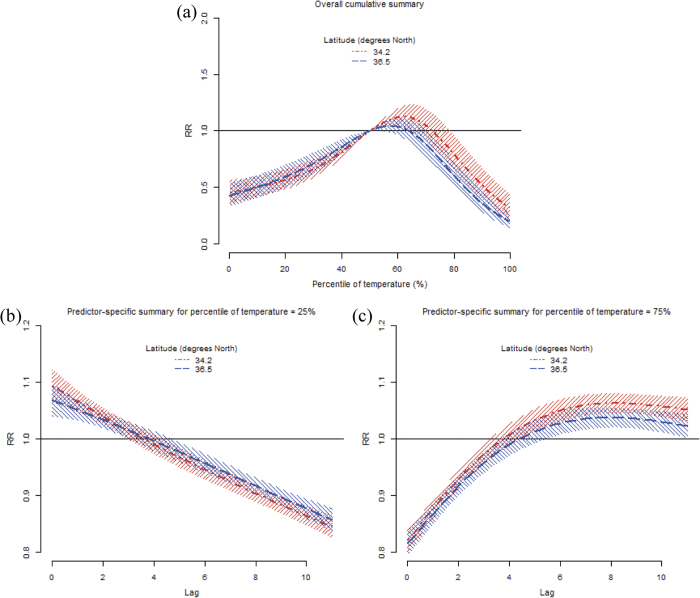
The pooled temperature-morbidity association by latitude in all 47 Japanese prefectures. Predictions for the 25^th^ (dot-dashed line) and 75^th^ (dashed line) percentiles of latitude from meta-regression for (**a**) overall cumulative summary, and predictor-specific summaries at (**b**) 25^th^ and (**c**) 75^th^ percentiles of temperature. Reference at 50^th^ percentile of temperature.

The results of the meta-regression including latitude are illustrated in Fig. 3. The top panel suggests a differential overall cumulative association between northern and southern prefectures. Overall, the evidence for a modifying effect was substantial, and the Wald test statistic was signficant (*p* < 0.001). Latitude accounted for heterogeneity among prefectures, with an *I*^*2*^ of 78.7% and a significant Cochran Q test statistic (*p* < 0.001). The bottom panels indicate an identical modifying effect in predictor-specific summary associations at the 25^th^ and 75^th^ temperature percentiles. The Wald test consistently indicated significance at higher (*p* = 0.002) and lower temperatures (*p* = 0.038). Cochran Q tests were also significant for the lag curve at the 25^th^ and 75^th^ temperature percentiles (*p* < 0.001), with *I*^*2*^ values of of 63.7% and 67.9%, respectively.

To investigate whether the results were sensitive to the level of control exercised for time trends, analyses were repeated using different degrees of freedoms. The estimated effects of temperature changed only marginally. Specifically, the model yielded a pooled overall RR values with respect to the incidence of infectious gastroenteritis of 1.15 (95% CI: 1.10, 1.20) with 1 df and 1.03 (95% CI: 1.00, 1.06) with 2 df. We also confirmed that the shapes of the pooled associations predicted in absolute temperature scale (Supplementary Figs S1–S3) were broadly similar to that predicted in relative temperature scale (Figs 1, 2, 3). The highest pooled overall RR was 1.52 (95% CI: 1.24, 1.85) at 16.3 °C.

## Discussion

This study produced several notable results; most importantly, the pooled overall risk of infectious gastroenteritis morbidity was highest around the 60^th^ percentile of temperature in all 47 prefectures. Moreover, the effects of lower temperature were immediate and disappeared after a few weeks, whereas the effects of higher temperatures were delayed but persisted for several weeks. Despite wide variation in climatic conditions, maximum-morbidity temperatures were observed at approximately the 60^th^ percentile in all 47 prefectures. These findings suggest that, to a certain extent, populations adapt to local weather conditions.

We observed a broadly inverted U-shaped association between temperature and morbidity, consistent with previous regional studies conducted in Fukuoka, Japan^7^,^8^, which also included a variety of weather-related variables, lag times, and statistical modelling techniques to control for time-varying confounders. However, such factors could alter temperature–morbidity associations between prefectures. By using consistent methods across all prefectures, we eliminated the possibility that comparisons could be confounded by methodological differences.

Using a pooled analysis, we observed that the overall national maximum-morbidity temperature corresponded to approximately the 60^th^ percentile, indicating that infectious gastroenteritis risk may be greatest at this percentile. We also confirmed that the inverted U-shapes of the pooled associations predicted in absolute temperature scale were broadly consistent with that predicted in relative temperature scale. These results suggest that the consistency of inverted U-shape associations between temperature and the risk of infectious gastroenteritis is remarkable, and provide the good evidence for the associations. Prefectures with colder temperatures were characterized by lower threshold temperatures, whereas those with hotter temperatures exhibited higher threshold temperatures. Differences in threshold temperature according to prefecture may reflect differences in socioeconomic, demographic, and geographical factors, in addition to weather patterns^14^. Our results suggest that populations may adapt to local weather patterns in physiological, behavioral, and technological terms.

We also observed that the effects of lower temperature were acute, whereas the effects of higher temperatures were delayed at the national level. This suggests that timely preventive measures could reduce morbidity risk at lower temperatures; measures allowing for several weeks of protection should be implemented to reduce morbidity risk at higher temperatures. The complex lag pattern that we observed could be due to differences in the pathogens causing infectious gastroenteritis. Although data on infectious gastroenteritis pathogens were not available for analysis, previous studies suggest an increased risk of both bacterial diarrhea during summer and viral diarrhea during winter^3^,^15^. A recent study also indicated that both high and low temperatures significantly affected childhood diarrhea intensity and duration^16^. Regarding the effects of lower temperatures, norovirus epidemics peak in winter, with fewer cases reported during the warm season^17^,^18^. Several studies have also reported that lower temperatures increase virus replication and survival as well as the transmission of viral diarrhea^3^,^19^. In contrast, higher temperatures increase the incidence of *Salmonella*, *Campylobacter*, and *Escherichia coli* infections^10^,^20^,^21^,^22^,^23^. At intermediate temperatures, populations may be exposed to numerous viral, bacterial, and parasitic pathogens^7^. We speculate that the complex lag effects as our results suggested would be related to these characteristics, however, the lag pattern difference between lower temperature effect and higher temperature effect suggests difference in the biological mechanism. Because the response of bacterial and viral pathogens to the effects of climate variability may differ, our data may only reflect responses to the most common causes of infectious gastroenteritis and therefore should be interpreted with caution. Although the exact mechanism remains unclear, these characteristics of infectious gastroenteritis highlight the need for further studies on the effects of weather variability on specific pathogen-induced infectious gastroenteritis.

Variation in the impact of temperature on morbidity may also be modified by social, environmental, and behavioral factors. For example, hot weather could result in certain behavioral patterns, such as higher water comsumption and less conscientious hygiene practices, thereby promoting diarrhea transmission^9^. Other studies have reported that food poisoning and electrolyte imbalance are more likely to occur during periods of persistent hot temperatures^24^,^25^,^26^. In cooler weather, people may spend more time indoors, with the resultant close proximity increasing the likelihood of person-to-person transmission^19^. Additionally, long-term lag effects could be related to the fact that temperature influences the extent of contamination in the food supply and distribution systems^20^,^22^. In future studies, more precise modeling should be employed to elucidate complex relationships between temperature and infectious gastroenteritis transmission at a national level.

In the meta-regression that included latitude, the modifying effect of this variable was substantial, particularly in more southerly prefectures. This spatial heterogeneity in the temperature–morbidity relationship could be explained in terms of differing degrees of acclimatization of populations to local weather conditions^27^. Additionally, the modifying effect of latitude could be due to variations in the epidemiological characteristics of the different population subgroups. Although we could not identify all of the factors that might modify the decline in morbidity, our data suggest that preventive measures should focus on preparing local people and using more appropriate adaptation measures to better respond to potential future temperature changes.

The present study has several limitations. First, not all cases of infectious gastroenteritis in the community are represented by surveillance data. Such under-reporting can occur anywhere in the reporting chain, i.e., from the initial decision of a patient not to seek health care to failure of medical personnel to record cases in the disease registry. However, we suggest that this did not result in substantial bias because the degree of under-reporting is unlikely to have varied over time. Second, the participating sentinel medical institutions were recruited on a voluntary basis. However, this did not undermine the validity of comparisons made over time, which represented the primary goal of this study. Third, we analyzed weekly data between 2000 to 2012; the modeling accuracy of these associations would improve commensurate with a longer study period or more detailed dataset. Fourth, there are many factors need to be considered in explanation of the heterogeneity between prefectures other than latitude, such as the proportion of people living in urban/rural environment; population density, the proportion of children under 5 year old in the population, education level, and living in poverty. For applying these variables as meta-predictors, further study would be important.

The results of this study have practical implications for public health officials. Elucidation of the relationship between climate variability and infectious gastroenteritis transmission is important for disease control and prevention. The present findings may facilitate public health officials’ ability to predict epidemics in each prefecture and to prepare for the effects of climatic change on infectious gastroenteritis. This could be achieved by implementing preventive public health measures, including forewarning medical staff, community-based campaigns, early-warning weather forecasts, and more efficiacious infectious gastroenteritis control programs. Our data suggest that local health departments in each prefecture should consider regional sensitivity to temperature when planning region-specific policies for disease control and prevention.

In conclusion, this study provides quantitative evidence that the temperatures associated with the highest morbidity in infectious gastroenteritis occur at approximately the 60^th^ percentile in all 47 Japanese prefectures. The effects of lower temperature were immediate but disappeared after a few weeks, whereas the effects of higher temperatures were delayed but persisted for several weeks. Public health strategies aimed at controlling temperature-related infectious gastroenteritis may be more effective when tailored to region-specific weather conditions.

## Methods

### Data collection

The number of infectious gastroenteritis cases is reported on a weekly basis using data from approximately 3,000 sentinel medical institutions in Japan under the Infectious Disease Control Law^7^,^8^. Infectious gastroenteritis is defined by clinical factors including sudden stomach ache, vomiting, and diarrhea. However, sentinel medical institutions are not required under this law to report information obtained from laboratory tests. We obtained clinical data recorded and reported by sentinel volunteers to the National Institute of Infectious Diseases, the national research institute of the Ministry of Health, Labour and Welfare, Japan. We also obtained data from the Japan Meteorological Agency on daily average temperature, relative humidity, and rainfall in 47 Japanese prefectures. Weekly means for average temperatures, relative humidity, and rainfall were calculated from daily records.

This study was approved by the ethics committee at Kyushu University Graduate School of Medical Sciences. The requirement for written informed consent was waived. Patient records and other patient information remained anonymous and de-identified prior to analysis. All methods were carried out in accordance with approved guidelines and regulations.

### Statistical analysis

We employed a two-stage analysis using time-series data from 47 Japanese prefectures to investigate temperature–morbidity associations at the national level. In the first stage, we used a time-series model for each prefectural dataset to estimate prefecture-specific temperature–morbidity relationships, allowing for nonlinearity and delayed effects. These estimated relationships were then pooled at the prefecture level in the second stage within a multivariate meta-analysis. The details of this approach have been documented elsewhere^11^,^28^.

The temperature–morbidity association in each prefecture is generally considered in the context of temperature measured using an absolute scale. However, temperature ranges differ among the 47 prefectures, so combining curves across prefectures using non-overlapping temperature ranges is problematic. Additionally, because several studies have indicated climate change adaptation among populations^29^,^30^, overall effects might be more reliable in terms of temperature percentiles than in terms of absolute temperature scales^31^. Therefore, we evaluated the overall effects of temperature on morbidity using relative temperature scales, and we standardized prefecture-specific temperatures to prefecture-specific percentiles. Results are expressed in terms of temperature percentiles, which correspond to different prefecture-specific temperatures. When curves on the relative temperature percentile scale are similar across prefectures, relative risk across percentiles is also similar. Conversely, when curves on the absolute scale of temperature are similar, they differ on the relative scale across prefectures with different climates.

### First-stage time series model

In the first stage of data analysis, we used a regression model to obtain prefecture-specific estimates assuming a quasi-Poisson distribution, thereby allowing for overdispersion. For each prefecture, we modelled the nonlinear and delayed effect of temperatures using distributed lag nonlinear models^11^,^28^. A flexible, cross-basis function was defined using a natural cubic spline for temperature space. We also defined a natural cubic spline with an intercept for the lag space, with a maximum lag time of up to 11 weeks. We set a natural cubic spline basis using 3 degrees of freedom each for temperature and lag. The choice of natural cubic spline was motivated by previous study^12^. An 11-week lag period was selected based on comparison of the quasi-likelihood Akaike Information Criteria (Q-AIC)^32^ of the models. The model with the lowest Q-AIC value was selected.

The prefecture-specific parameters of the cross-basis function expressed the nonlinear and delayed temperature–morbidity association of each prefecture, which was then reduced to three summaries expressing the overall cumulative exposure–response relationship and the lag-response association specific to the 25^th^ and 75^th^ percentiles. The latter two summaries represent the lag pattern of lower and higher temperatures, respectively. Reduction was performed for each summary by computing transformed parameters for the unidimensional natural cubic splines for temperature or lag space using the original parameters of the cross-basis delineated above. This method is described in more detail elsewhere^11^.

The prefecture-specific Poisson time-series model is described by the following equation (1):
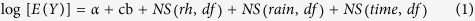
where *E*(*Y*) represents expected weekly infectious gastroenteritis incidence, cb represents the cross basis matrix for weekly mean temperature, *NS* (*rh*, *df*  ) represents the natural cubic spline of relative humidity, and df is the degrees of freedom for relative humidity; 3 df was used to control for the effects of relative humidity. *NS* (*rain*, *df*  ) represents the natural cubic spline of rainfall, with 3 df used to control for the effects of rainfall; and *NS* (*time*, *df*  ) represents the natural cubic spline of time, with 3 df per year used to control for the effects of seasonality and long-term trends. The choice of 3 df for relative humidity, rainfall, and seasonality and long-term trends was motivated by previous studies^7^,^8^,^33^. The model was described in Supplementary Methods S1.

### Second-stage meta-analysis

In the second stage of analysis, a multivariate meta-analysis was applied to pool the three sets of prefecture-specific estimates obtained following the reduction of the first stage in order to evaluate the nonlinear temperature–morbidity relationship at the national level^11^. Multivariate meta-analyses were applied to examine national, pooled estimates using a random effects model according to maximum likelihood. Heterogeneity was evaluated by a multivariate extension of the *I*^*2*^ index, which quantified the proportion of variability due to true differences across prefectures.

For sensitivity analysis, weather**–**morbidity relationships were also estimated using different degrees of freedom for time trends (1 and 2 df per year). In addition, we also estimated the relationships between temperature and morbidity with temperature as Celsius degree. All analyses were conducted using the R software package (ver. 3.1.2; R Core Team, R Foundation for Statistical Computing, Vienna, Austria).

## Additional Information

**How to cite this article**: Onozuka, D. and Hagihara, A. Nationwide variation in the effects of temperature on infectious gastroenteritis incidence in Japan. *Sci. Rep.* **5**, 12932; doi: 10.1038/srep12932 (2015).

## Supplementary Material

Supplementary Information
