# Supplementary material for: Nationwide variation in the effects of temperature on infectious gastroenteritis incidence in Japan
Source: Sci Rep. 2015 Aug 10;5:12932. doi: 10.1038/srep12932 (PMC4530438; doi:10.1038/srep12932)
Supplement: Supplementary Information [file srep12932-s1.pdf]

Supplementary information

Nationwide variation in the effects of temperature on infectious gastroenteritis incidence in Japan

Daisuke Onozuka[1], Akihito Hagihara[1]

[1] Department of Health Communication, Kyushu University Graduate School of Medical Sciences, Fukuoka, Japan

Supplementary Table S1. Summary statistics for morbidity, weather and latitude for 47 prefectures in Japan from 2000 to 2012.

Code	Prefecture	Latitute	Population (2010)	Average no. of cases	Average temperature (°C)	Average humidity (%)	Average rainfall (mm)
1	Hokkaido	43.06	5,506,419	391.8	9.3	68.5	3.1
2	Aomori	40.82	1,373,339	136.9	10.7	74.5	3.9
3	Iwate	39.70	1,330,147	161.7	10.6	73.3	3.5
4	Miyagi	38.27	2,348,165	463.9	12.8	71.3	3.5
5	Akita	39.72	1,085,997	188.6	12.1	72.6	4.5
6	Yamagata	38.24	1,168,924	214.0	12.0	74.0	3.3
7	Fukushima	37.75	2,029,064	288.5	13.3	68.8	3.3
8	Ibaraki	36.34	2,969,770	312.5	14.0	72.7	3.8
9	Tochigi	36.57	2,007,683	207.9	14.3	68.8	4.2
10	Gunma	36.39	2,008,068	369.8	14.9	60.9	3.5
11	Saitama	35.86	7,194,556	1154.4	15.4	63.9	3.5
12	Chiba	35.61	6,216,289	794.7	16.2	68.2	4.1
13	Tokyo	35.69	13,159,388	1021.3	16.6	59.5	4.4
14	Kanagawa	35.45	9,048,331	1265.6	16.1	64.7	4.8
15	Niigata	37.90	2,374,450	383.8	14.1	70.5	5.0
16	Toyama	36.70	1,093,247	235.2	14.5	77.2	6.5
17	Ishikawa	36.59	1,169,788	226.4	15.0	70.0	6.4
18	Fukui	36.07	806,314	233.8	14.7	74.5	6.3
19	Yamanashi	35.66	863,075	106.9	15.0	62.7	3.3
20	Nagano	36.65	2,152,449	362.2	12.1	71.1	2.6
21	Gifu	35.39	2,080,773	194.0	16.1	65.1	4.9
22	Shizuoka	34.98	3,765,007	596.3	16.8	68.0	6.5
23	Aichi	35.18	7,410,719	1015.2	16.1	64.8	4.3
24	Mie	34.73	1,854,724	391.1	16.2	65.8	4.3
25	Shiga	35.00	1,410,777	169.0	15.0	73.9	4.4
26	Kyoto	35.02	2,636,092	450.1	16.1	64.1	4.0
27	Osaka	34.69	8,865,245	1114.4	17.1	62.8	3.5
28	Hyogo	34.69	5,588,133	919.7	17.0	65.1	3.3
29	Nara	34.69	1,400,728	184.1	15.1	72.2	3.6
30	Wakayama	34.23	1,002,198	165.8	16.8	64.1	3.8
31	Tottori	35.50	588,667	159.7	15.1	72.8	5.3
32	Shimane	35.47	717,397	160.6	15.2	74.1	4.8
33	Okayama	34.66	1,945,276	349.3	16.5	65.5	3.0
34	Hiroshima	34.40	2,860,750	497.2	16.5	67.1	4.0
35	Yamaguchi	34.19	1,451,338	407.7	15.8	69.9	5.0
36	Tokushima	34.07	785,491	136.8	16.8	65.9	4.4
37	Kagawa	34.34	995,842	193.6	16.8	65.4	3.0
38	Ehime	33.84	1,431,493	326.8	16.8	65.2	3.7
39	Kochi	33.56	764,456	161.4	17.4	68.7	7.1

40	Fukuoka	33.61	5,071,968	944.0	17.4	65.4	4.4
41	Saga	33.25	849,788	132.4	16.9	67.5	5.0
42	Nagasaki	32.74	1,426,779	249.7	17.5	68.6	4.9
43	Kumamoto	32.79	1,817,426	384.2	17.4	68.0	5.4
44	Oita	33.24	1,196,529	401.1	16.9	66.7	4.7
45	Miyazaki	31.91	1,135,233	412.2	17.8	72.3	7.1
46	Kagoshima	31.56	1,706,242	398.4	18.9	68.0	6.2
47	Okinawa	26.21	1,392,818	70.8	23.3	72.4	6.1

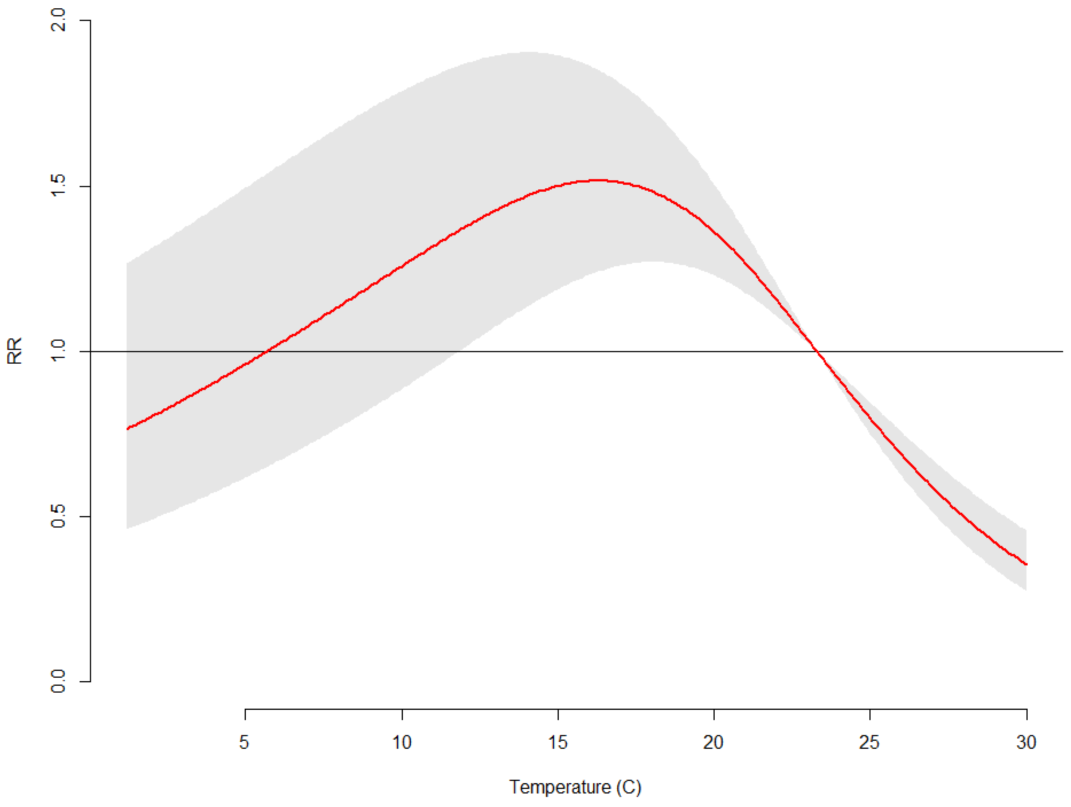

Supplementary Figure S1. The pooled overall cumulative temperature-morbidity association in all 47 Japanese prefectures. Reference at 23.3°C.

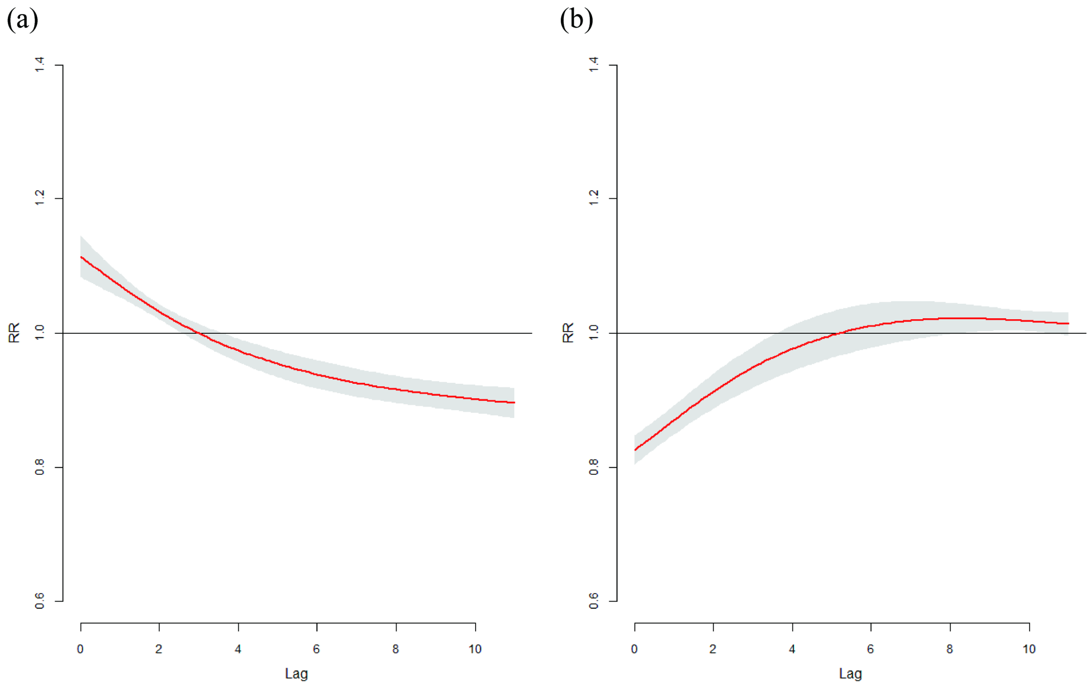

Supplementary Figure S2. The pooled predictor-specific temperature-morbidity association in all 47 Japanese prefectures. The pooled (95% CI as grey area) summaries at (a) 8.1°C (25th percentile) and (b) 22.7°C (75th percentile). Reference at 23.3°C.

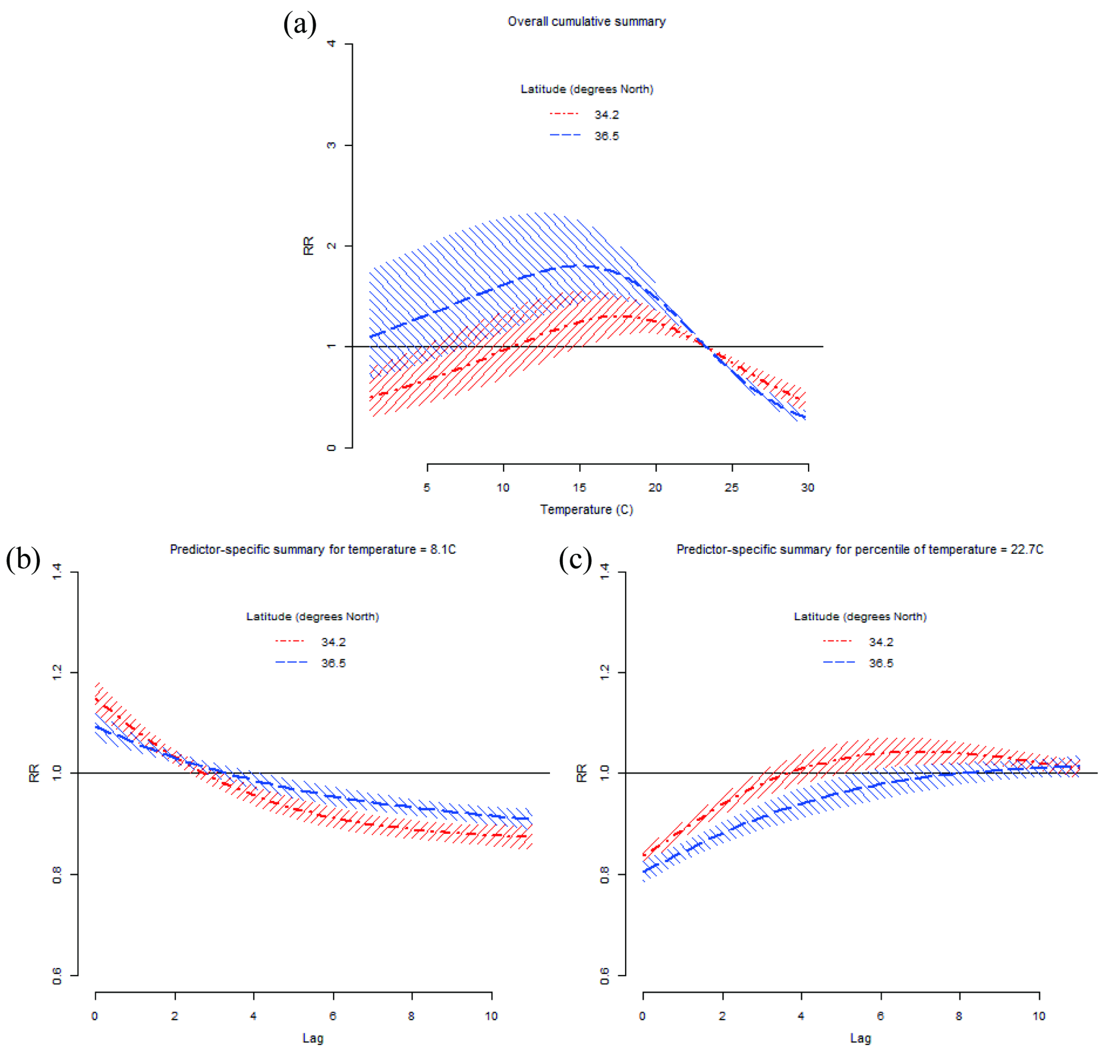

Supplementary Figure S3. The pooled temperature-morbidity association by latitude in all 47 Japanese prefectures. Predictions for the 25th (dot-dashed line) and 75th (dashed line) percentiles of latitude from meta-regression for (a) overall cumulative summary, and predictor-specific summaries at (b) 8.1°C (25th percentile) and (c) 22.7°C (75th percentile). Reference at 23.3°C.

Supplementary Methods S1. R code to study time series.

To analyze the effect of temperature on infectious gastroenteritis cases, the following codes were used.

```
#Analysis using distributed lag non-linear models:
lag <- c(0,11)
bound <- colMeans(ranges)
varknots <- equalknots(bound,fun="ns",df=3)
lagknots <- logknots(11,df=3,int=T)
argvar <- list(fun="ns",df=3,knots=varknots,bound=bound)
arglag <- list(fun="ns",knots=lagknots)
cb <- crossbasis(sub$ptmean,lag=lag,argvar=argvar,arglag=arglag)
model <- glm(case ~ cb+ns(prh,df=3)+ns(prain,df=3)+ns(time,df=3*13), family=
quasipoisson(),sub)
```

where:

case is the number of weekly cases,

cb is a variable indicating cross basis matrix for percentile of weekly mean temperature,

prh is a variable indicating percentile of weekly mean humidity,

prain is a variable indicating percentile of weekly mean rainfall,

time is a variable indicating time to control for the effects of seasonality and long-term trends.
